# *N*-Benzyl-(2,5-dioxopyrrolidin-1-yl)propanamide (AS-1) with Hybrid Structure as a Candidate for a Broad-Spectrum Antiepileptic Drug

**DOI:** 10.1007/s13311-019-00773-w

**Published:** 2019-09-04

**Authors:** Krzysztof Kamiński, Katarzyna Socała, Mirosław Zagaja, Marta Andres-Mach, Michał Abram, Marcin Jakubiec, Mateusz Pieróg, Dorota Nieoczym, Anna Rapacz, Kinga Gawel, Camila V. Esguerra, Gniewomir Latacz, Annamaria Lubelska, Bartłomiej Szulczyk, Aleksandra Szewczyk, Jarogniew Jacek Łuszczki, Piotr Wlaź

**Affiliations:** 1 Jagiellonian University Medical College, Faculty of Pharmacy, Department of Medicinal Chemistry, Medyczna 9, 30-688 Cracow, Poland; 2 Department of Animal Physiology, Institute of Biology and Biochemistry, Faculty of Biology and Biotechnology, Maria Curie-Skłodowska University, Akademicka 19, 20-033 Lublin, Poland; 3 Isobolographic Analysis Laboratory, Institute of Rural Health, Jaczewskiego 2, 20-090 Lublin, Poland; 4 Jagiellonian University Medical College, Faculty of Pharmacy, Department of Pharmacodynamics, Medyczna 9, 30-688 Cracow, Poland; 5 Chemical Neuroscience Group, Centre for Molecular Medicine Norway, University of Oslo, Gaustadalléen 21, Forskningsparken, 0349 Oslo, Norway; 6 Department of Experimental and Clinical Pharmacology, Medical University of Lublin, Jaczewskiego 8b, 20-090 Lublin, Poland; 7 Jagiellonian University Medical College, Faculty of Pharmacy, Department of Technology and Biotechnology of Drugs, Medyczna 9, 30-688 Cracow, Poland; 8 Department of Drug Technology and Pharmaceutical Biotechnology, Medical University of Warsaw, Banacha 1, 02-097 Warsaw, Poland; 9 Laboratory of Physiology and Pathophysiology, Centre for Preclinical Research and Technology, Medical University of Warsaw, Banacha 1B, 02-097 Warsaw, Poland; 10 Department of Pathophysiology, Medical University of Lublin, Jaczewskiego 8b, 20-090 Lublin, Poland

**Keywords:** Drug-resistant epilepsy, PTZ-kindling model of epilepsy, isobolographic studies, electrophysiology, ADME-Tox properties, zebrafish

## Abstract

In our recent studies, we identified compound *N*-benzyl-2-(2,5-dioxopyrrolidin-1-yl)propanamide (AS-1) as a broad-spectrum hybrid anticonvulsant which showed potent protection across the most important animal acute seizure models such as the maximal electroshock (MES) test, the subcutaneous pentylenetetrazole (s.c. PTZ) test, and the 6-Hz (32 mA) test in mice. Therefore, AS-1 may be recognized as a candidate for new anticonvulsant effective in different types of human epilepsy with a favorable safety margin profile determined in the rotarod test in mice. In the aim of further pharmacological evaluation of AS-1, in the current study, we examined its activity in the 6-Hz (44 mA) test, which is known as the model of drug-resistant epilepsy. Furthermore, we determined also the antiseizure activity in the kindling model of epilepsy induced by repeated injection of pentylenetetrazole (PTZ) in mice. As a result, AS-1 revealed relatively potent protection in the 6-Hz (44 mA) test, as well as delayed the progression of kindling induced by repeated injection of PTZ in mice at doses of 15 mg/kg, 30 mg/kg, and 60 mg/kg. Importantly, the isobolographic analysis showed that a combination of AS-1 and valproic acid (VPA) at the fixed ratio of 1:1 displayed a supra-additive (synergistic) interaction against PTZ-induced seizures in mice. Thus, AS-1 may be potentially used in an add-on therapy with VPA. Moreover, incubation of zebrafish larvae with AS-1 substantially decreased the number, cumulative but not the mean duration of epileptiform-like events in electroencephalographic assay. Finally, the *in vitro* ADME-Tox studies revealed that AS-1 is characterized by a very good permeability in the parallel artificial membrane permeability assay test, excellent metabolic stability on human liver microsomes (HLMs), no significant influence on CYP3A4/CYP2D6 activity, and moderate inhibition of CYP2C9 in a concentration of 10 μM, as well as no hepatotoxic properties in HepG2 cells (concentration of 10 μM).

**Electronic supplementary material:**

The online version of this article (10.1007/s13311-019-00773-w) contains supplementary material, which is available to authorized users.

## Introduction

Epilepsy, which affects about 1% of the world population, belongs to the most common, heterogeneous, and debilitating neurological diseases with a high risk of drug resistance [1]. According to the International League Against Epilepsy, the current classification of seizure types distinguishes three major groups, as follows: generalized-onset seizures (with retained or impaired awareness), focal-onset seizures, and unknown-onset seizures [2]. Due to multifactorial pathomechanism and different clinical manifestations of epilepsy, about 30% of patients do not successfully respond to pharmacotherapy and are considered to have drug-resistant epilepsy (DRE) [3]. The refractory epilepsy usually requires the application of two or three various antiepileptic drugs (AEDs), preferentially with different mechanisms of action. Unfortunately, this therapeutic regimen increases the risk of drug–drug interactions (DDIs), and may also lead to potentiation of side effect and, finally, to discontinuation of the treatment. Patients suffering from uncontrolled seizures are prone to numerous and serious psychiatric conditions such as depression, cognitive impairment, or anxiety [4, 5]. Furthermore, current pharmacotherapy is only symptomatic, as the available AEDs inhibit seizures but these medications are devoid of antiepileptogenic, as well as disease-modifying properties. As a result, both old- and new-generation AEDs do not cure epilepsy and therefore cannot be used as prophylaxis especially of idiopathic disease. Bearing in mind the aforementioned facts, the search for new AEDs is still hugely necessary and should be directed on broad-spectrum anticonvulsants, preferentially with complex mechanisms of action and favorably with antiepileptogenic and/or disease-modifying properties [6, 7].

**Fig. 1:**
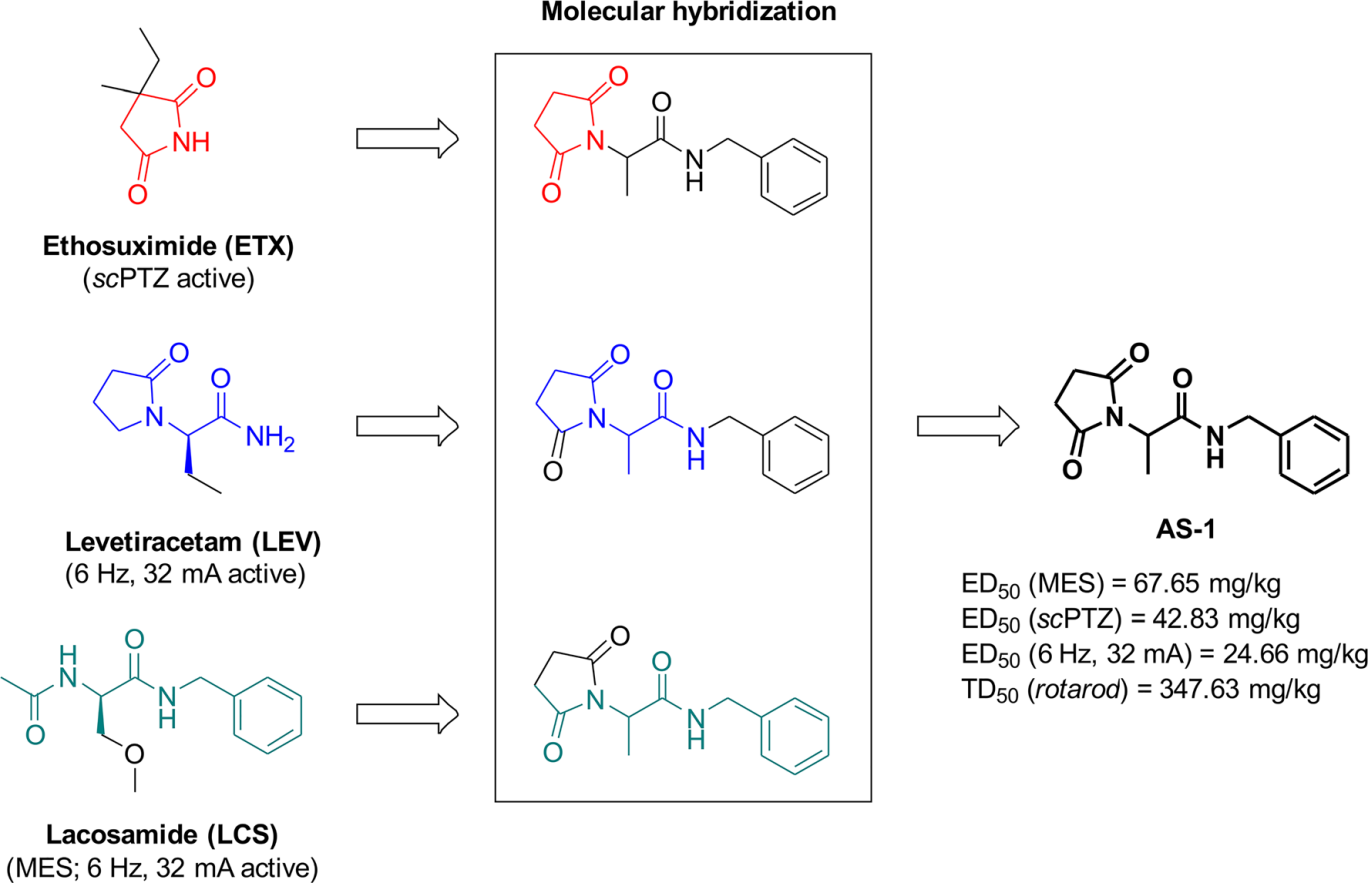
Molecular hybridization which yielded the compound AS-1. Anticonvulsant data in mice injected i.p.

With the aim of obtaining new and effective anticonvulsants, since many years, our efforts have been focused on the development of hybrid compounds based on the pyrrolidine-2,5-dione core fragment [8–14]. It should be stressed that in recent years, the hybrid substances are of great importance in designing of multitargeted compounds, as they have been proven to be advantageous in the treatment of multifactorial diseases and also have been proven to alleviate health conditions linked to drug resistance [15, 16]. Epilepsy without any doubt fulfills both the aforementioned criteria. Multitargeted compounds, proposed by our team, were designed as integrated hybrids which combine structural fragments of known and therapeutically relevant AEDs, such as ethosuximide (ETX, pyrrolidine-2,5-dione derivative), levetiracetam (LEV, pyrrolidin-2-one derivative), and lacosamide (LCS, classified as functionalized amino acid). The applied molecular hybridization yielded compounds with potent and wide anticonvulsant properties in the preclinical studies, as they were effective in the maximal electroshock (MES) seizure test, the 6-Hz (32 mA) seizure model, and the subcutaneous pentylenetetrazole (s.c. PTZ) seizure model [8–14]. Importantly, compounds with the aforementioned profile in the *in vivo* studies may be effective in the pharmacotherapy of a wide range of human epilepsies including tonic–clonic seizures (with or without secondary generalization), absence epilepsy, as well as myoclonic and partial seizures. Furthermore, the results proved the success of proposed molecular hybridization, as our compounds revealed wider anticonvulsant activity than each individual AED, which creates hybrid structure as follows: ETX (s.c. PTZ active), LEV (6-Hz, 32 mA active), and LCS (effective in the MES and 6-Hz (32 mA) models). Among the obtained substances, the favorable pharmacological and toxicological profile was observed for *N*-benzyl-2-(2,5-dioxopyrrolidin-1-yl)propanamide (AS-1), which was identified as one of the lead compounds (Fig. 1).

The preclinical data for AS-1 (Fig. 1) revealed more potent protection in each seizure model (MES, s.c. PTZ, 6-Hz) and simultaneously distinctly more pronounced separation between anticonvulsant and neurotoxic doses compared to valproic acid (VPA), which was tested in the same experimental conditions as follows: median effective dose (ED_50_; MES) = 252.74 mg/kg, ED_50_ (s.c. PTZ) = 239.45 mg/kg, ED_50_ (6-Hz, 32 mA) = 130.65 mg/kg, and TD_50_ (rotarod test) = 430.77 mg/kg (data in mice injected intraperitoneally (i.p.) from [10, 12]). It is noteworthy, despite of implementation to pharmacotherapy many new AEDs, VPA is still recognized as the clinically relevant wide-spectrum AED with complex mechanisms of action and a wide range of indications in different types of human epilepsy.

The mouse 6-Hz model applying the 44 mA stimulus intensity has been identified as a useful tool in the identification of novel compounds with potential efficacy against drug-resistant epilepsy [17, 18]. Thus, in the current studies, AS-1 was screened in the 6-Hz (44 mA) test. Furthermore, taking into consideration the favorable activity of AS-1 in the acute models of seizures, we have determined herein its protective properties in the pentylenetetrazole (PTZ)-induced chemical kindling model. In this chronic model of epilepsy, repeated injection of PTZ evokes progressive enhancement of seizure susceptibility [19]. Moreover, the effects of repeated treatment with AS-1 on kindling-associated behavioral despair and anxiety were evaluated. Using the timed intravenous (i.v.) PTZ seizure threshold model, we determined the changes in seizure susceptibility following PTZ kindling. In addition, acute effects of AS-1 on seizure threshold in naïve animals were also investigated.

In the last few years, we can observe a tendency to study potential and clinically beneficial DDIs, mainly due to resistance to one drug treatment (i.e., in case of refractory epilepsy). It turns out that two or more drugs given in combination may produce effect more potent or weaker than that observed for individual agents. Therefore, much is being done to find the most favored combinations. It should be stressed here the treatment with more than one drug may cause more side effects as well. Isobolographic analysis is a method enabling assessment of the interaction between drugs and allows their classification as supra-additive (synergistic), additive, sub-additive (antagonistic), and indifferent (neutral) [20–22]. Based on the results obtained from our previous studies indicating marked anticonvulsant properties of AS-1 in various animal models of seizures [10, 12], we decided to determine the interaction between AS-1 and one of the most common classic AEDs—VPA, using isobolographic analysis in the PTZ model of seizures. Additionally, we evaluated the impact of the AS-1 + VPA combination on the motor coordination, muscular strength, and long-term memory in mice. We also investigated the effect of 24 h-long incubation with AS-1 on the number and duration of electroencephalographic (EEG) epileptiform-like discharges induced by acute PTZ administration in 7-day post-fertilization zebrafish larvae.

In the present study, we also attempted to discuss the mechanism of action of AS-1, which remains unknown. Finally, bearing in mind drug-like properties of AS-1, several ADME-Tox parameters were determined in *in vitro* assays.

## Material and Methods

### Compounds

AS-1 was obtained in the Department of Medicinal Chemistry, Jagiellonian University Medical College, according to the procedures described elsewhere [10]. The reference AED sodium valproate (VPA) and pentylenetetrazole (PTZ) were purchased from a commercial supplier (Sigma-Aldrich, St. Louis, MO). Before *in vivo* and *in vitro* studies, AS-1 was fully characterized using the spectral (^1^H NMR, ^13^C NMR) and elemental (C, H, N) analyses. The purity and homogeneity of the compounds were assessed by thin-layer chromatography (TLC) and the gradient ultra-performance liquid chromatography (UPLC). TLC was performed on silica gel 60 F_254_ precoated aluminum sheets (Macherey-Nagel, Düren, Germany), using the following developing system: dichloromethane/methanol, 9:0.3 (*v*/*v*). A spot was detected by its absorption under UV light (*λ* = 254 nm). The UPLC analysis and mass spectra (LC-MS) were obtained on Waters ACQUITY TQD system (Waters, Milford, CT) with the MS-TQ detector and UV-vis-DAD eλ detector. The ACQUITY UPLC BEH C18 1.7-μm (2.1 mm × 100 mm) column was used with the VanGuard ACQUITY UPLC BEH C18, 1.7 μm (2.1 × 5 mm) (Waters). Standard solutions (1 mg/ml) were prepared in analytical grade MeCN/water mixture (1:1; *v*/*v*). Conditions applied were as follows: eluent A (water/0.1% HCOOH), eluent B (MeCN/0.1% HCOOH), a flow rate of 0.3 ml/min, a gradient of 5–100% B over 10 min, and an injection volume of 10 μl. The UPLC retention time (*t*_R_) is given in minutes. Elemental analysis (C, H, and N) for AS-1 was carried out by a micro method using the Vario EI III elemental analyzer (Hanau, Germany). The results of elemental analyses were within ± 0.4% of the theoretical values. ^1^H NMR and ^13^C NMR spectra were obtained in a JEOL 500 (JEOL USA, Inc., MA), in CDCl_3_ operating at 500 MHz (^1^H NMR) and 126 MHz (^13^C NMR). Chemical shifts are reported in *δ* values (ppm) relative to TMS *δ* = 0 (^1^H), as internal standard. The *J* values are expressed in hertz (Hz). Signal multiplicities are represented by the following abbreviations: s (singlet), br. s (broad singlet), d (doublet), q (quartet), and m (multiplet).The detailed physicochemical and spectral data for AS-1 are summarized in Table S1.

### *In Vivo* Studies in Mice

#### Animals

All experiments were performed on male Swiss albino mice (weighing 22–30 g) obtained from a licensed breeder. Animals were housed in groups of 7–8 per cage under controlled environmental conditions (temperature 21–24 °C, relative humidity 45–65%) at 12 h light–dark cycle (lights on at 6 a.m.) with free access to standard laboratory chow and tap water. Before being used in the experiments, mice were allowed to adapt to the laboratory conditions for at least 1 week. Housing and experimental procedures were conducted under the guidelines provided by the European Union Directive of 22 September 2010 (2010/63/EU) and Polish legislation concerning animal experimentation. All *in vivo* procedures were approved by the Local Ethical Committee in Lublin (License Nos. 16/2017 and 6/2019) and by the Local Ethical Committee in Cracow (License No. 149/2018).

#### Treatment

For PTZ kindling, AS-1 was suspended in a 0.5% aqueous solution of methylcellulose (tylose), while VPA and PTZ were dissolved in normal saline. AS-1 and VPA were injected repeatedly every 24 h for 33 consecutive days. In studies assessing the acute effect of AS-1 on the seizure threshold, AS-1 and VPA were given 30 min and 15 min before the test, respectively. In isobolographic studies, AS-1 (suspended in a 1% Tween 80) and VPA were administered 30 min before s.c. injection of PTZ. All suspensions and solutions were prepared freshly and administered in a constant volume of 0.1 ml per 10 g of body weight. In all experiments, AS-1 and VPA were injected i.p. Control animals received vehicles only.

#### PTZ Kindling

For kindling induction, mice were repeatedly injected i.p. with PTZ at a subconvulsive dose of 40 mg/kg on every other day for 33 days (17 injections). After each PTZ injection, mice were placed separately into transparent cages for 30 min for behavioral observation to assign an appropriate seizure score. Seizure severity was categorized according to a modified Racine’s scale [23] as follows: stage 0, no change in behavior; stage 1, immobility and ear and facial twitching; stage 2, myoclonic jerks; stage 3, forelimb clonus; stage 4, clonic seizure with rearing; stage 5, generalized clonic seizure with loss of righting reflex; and stage 6, fore limb and hind limb tonus. After each PTZ injection, the mean seizure severity score for each experimental group was calculated. Non-kindled (control) mice were treated as kindled animals, except that they were injected with normal saline instead of PTZ.

Twenty-four hours after the last PTZ injection (on day 34), mice were subjected to the locomotor activity test, the elevated plus maze test, and the forced swim test. Forty-eight hours after the last PTZ injection (on day 35) the i.v. PTZ seizure threshold test was performed.

Experimental grouping for PTZ kindling was as follows: (1) normal control, 0.5% tylose (33 injections) + saline (17 injections); (2) PTZ control, 0.5% tylose (33 injections) + PTZ (17 injections); (3) positive control, VPA at 150 mg/kg (33 injections) + PTZ (17 injections); (4) AS-1 at 15 mg/kg (33 injections) + PTZ (17 injections); (5) AS-1 at 30 mg/kg (33 injections) + PTZ (17 injections); and (6) AS-1 at 60 mg/kg (33 injections) + PTZ (17 injections).

#### Locomotor Activity Test

Spontaneous locomotor activity of mice was monitored using the IR Actimeter system supported by SedaCom32 software (Panlab/Harvard Apparatus, Barcelona, Spain) according to the method described in detail elsewhere [13].

#### Elevated Plus Maze Test

The elevated plus maze test was used to assess the anxiety-like behavior in kindled animal, as described in detail in our previous study [13].

#### Forced Swim Test

The forced swim test was used to assess the depressive-like behavior in kindled animals. The procedure was performed as described in detail elsewhere [13].

#### i.v. PTZ Seizure Threshold Test

The timed i.v. PTZ test was used to evaluate the changes in seizure threshold in both naïve and kindled mice. The experimental procedure has been described in detail in our earlier studies [13].

#### The 6-Hz Seizure Model (Current Intensity of 44 mA)

The 6-Hz-induced seizures were elicited by corneal stimulation (6 Hz, 44 mA, 0.2 ms rectangular pulse width, 3 s duration) using a constant-current device (ECT Unit 57800; Ugo Basile, Gemonio, Italy) as described previously [14]. A drop of 1% solution of lidocaine hydrochloride was applied to the mouse corneas before stimulation to provide local anesthesia and ensure optimal current conductivity. After the electrical stimulation, mice were gently released and observed for the presence or absence of seizure activity, being characterized by immobility or stun posture associated with rearing, forelimb clonus, twitching of the vibrissae, and Straub tail. Mice resuming normal behavior within 10 s after stimulation were considered as protected [24]. Subsequently, for compounds tested (AS-1 and VPA), the ED_50_ value, which is defined as the dose of a drug protecting 50% of animals against seizures, was determined. To evaluate the ED_50_ value, three groups of 8 mice were injected with various doses of the tested compound. The log-probit method was applied to statistically determine the ED_50_ values, which are accompanied by their respective 95% confidence limits [25].

#### s.c. PTZ-Induced Convulsions

The anticonvulsant activity of AS-1 and VPA against PTZ-induced clonic seizures was determined after s.c. administration of PTZ at a dose of 100 mg/kg [26, 27]. Following PTZ administration, mice were placed separately in transparent Plexiglas cages (25 cm × 15 cm × 10 cm) and observed for 30 min for the occurrence of clonic seizures. Clonic seizure activity was defined as the clonus of the whole body lasting for over 3 s, with an accompanying loss of righting reflex. The number of animals convulsing out of the total number of mice tested was noted for each treatment regimen. The animals were administered with increasing doses of VPA and AS-1, and the anticonvulsant activity of each drug was evaluated as the ED_50_ value (median effective dose of the drug, which protects 50% of mice against clonic convulsions) calculated from the respective log-probit dose–response relationship line according to Litchfield and Wilcoxon [25]. The anticonvulsant activity of AS-1 alone was studied at doses of 50–100 mg/kg, whereas that of VPA alone at doses of 125–200 mg/kg against the clonic phase of the PTZ-induced seizures in mice. Similarly, the anticonvulsant activity of a mixture of AS-1 with VPA was evaluated and expressed as ED_50 mix_, corresponding to the dose of a mixture of both drugs required to protect 50% of animals tested against PTZ-induced clonic convulsions.

#### Isobolographic Analysis of Interactions

Interactions between AS-1 and VPA against PTZ-induced seizures were analyzed according to the methodology previously detailed in earlier studies [26, 28, 29]. In the present study, the isobolographic analysis comprised of 5 stages, as follows:

1. Determination of ED_50_ values for AS-1 and VPA (administered separately) by means of log-probit linear regression analysis according to Litchfield and Wilcoxon [25].
2. Calculation of purely additive ED_50 add_ values ± SEM for a mixture of the examined combination of AS-1 and VPA at the fixed ratio 1:1. The ED_50 add_ represents a total additive dose of the drugs in the mixture, providing theoretically a 50% protection against PTZ-induced seizures.
3. Experimental determination of the ED_50 mix_ values ± SEM for the combination of AS-1 and VPA at the fixed ratio 1:1. ED_50 mix_ is an experimentally determined total dose of a mixture of two component drugs, administered at a fixed ratio combination sufficient for the 50% protective effect against PTZ-induced seizures. To determine the ED_50 mix_ value, both drugs in the mixture (at proportionally raised doses) were administered to the mice and a dose–response relationship for the mixture was denoted using Litchfield and Wilcoxon [25]’s log-probit method.
4. Statistical comparison of the experimentally derived ED_50 mix_ values with their corresponding theoretically additive ED_50 add_ values was undertaken using the unpaired Student’s *t* test, according to Tallarida [30].
5. Graphical illustration of the examined interactions as isobolograms (i.e., simple forms of interaction visualizations).

#### Chimney Test

The effects of AS-1, VPA, and their combination (at the fixed ratio 1:1 from the PTZ test) on motor coordination impairment were quantified with Boissier et al. [31]’s chimney test. The pretreatment time for AS-1 and VPA in the chimney test was identical to that of the PTZ test. This experimental procedure has been described in detail in our earlier studies [26, 32].

#### Grip Strength Test

The effects of AS-1, VPA, and their combination (at the fixed ratio 1:1 from the PTZ test) on muscular strength in mice were quantified by the grip strength test. The time before the commencement of the grip strength test (after drug administration) was identical to that of the PTZ test. This experimental procedure has been described in detail in our earlier studies [26, 32].

#### Passive Avoidance Task

To assess potential acute adverse effects of AS-1, VPA, and their combination (at the fixed ratio 1:1 from the PTZ test) on the mice’s ability to acquire the task (learning) and to recall the task (retrieval), the passive avoidance test was used as described in detail in our previous study [26, 32].

### EEG Recordings in Zebrafish Larvae

#### Animals

Adult zebrafish (*Danio rerio*) stocks of AB strain (Zebrafish International Resource Center, Eugene, OR) were maintained at standard aquaculture conditions, i.e., 28.5 °C, on a 14 h/10 h light/dark cycle, lights on at 8 a.m. Fertilized eggs were collected via natural spawning. Embryos were reared under constant light conditions in embryo medium, i.e., Danieau’s buffer (1.5 mM HEPES, pH 7.6, 17.4 mM NaCl, 0.21 mM KCl, 0.12 mM MgSO_4_, and 0.18 mM Ca(NO_3_)_2_). All embryos and larvae were kept in an incubator, at 28.5 °C. For EEG experiments, larvae of 6 days post fertilization (dpf) were used. The experiment was approved by the Norwegian Food Safety Authority experimental animal administration’s supervisory and application system (“Forsøksdyrforvatningen tilsyns- og søknadssystem”; FOTS 18/106800-1).

#### Treatment

Substance AS-1 was dissolved in DMSO in order to make a stock. Next, stock was diluted in embryo medium to reach a maximum 1% *w*/*v* DMSO in final solution. Embryo medium with an equivalent amount of DMSO was used as a vehicle control. PTZ was purchased from Sigma-Aldrich and dissolved to 80 mM (4× stock) in embryo medium.

#### Toxicological Assessment

Maximum tolerated concentration was evaluated prior to further experiments. Briefly, 4-dpf zebrafish larvae (*n* = 12/group) were incubated with a range of AS-1 doses at 28.5 °C for 18 h. The following parameters were scored after 2 h and 18 h of exposure: touch response, posture, edema, morphology, signs of necrosis, swim bladder, and heartbeat. The dose of 5 mM was chosen for the EEG experiment.

#### EEG Discharge Assessment

A single 6-dpf zebrafish larvae were placed in a 48-well plate (one larva per well) filled with 300 μl of vehicle or 300 μl of AS-1 solution. Subsequently, larvae were incubated for 20 h, at 28.5 °C. After incubation, larvae were exposed to vehicle or 20 mM PTZ for 5 min. Next, larvae were immobilized in a thin layer of 2% low-melting-point agarose and the glass electrode (resistance 1–5 MΩ) filled with artificial cerebrospinal fluid (124 mM NaCl, 2 mM KCl, 2 mM MgSO_4_, 2 mM CaCl_2_, 1.25 mM KH_2_PO_4_, 26 mM NaHCO_3_, 10 mM glucose) was placed into the optic tectum (MultiClamp 700B amplifier, Digidata 1440A digitizer; Molecular Devices, San Jose, CA) [33, 34]. Single recordings for each larva were performed for a period of 20 min. The discharges were analyzed according to the duration of spiking paroxysms, and only those were taken into account when the amplitude exceeded three times the background noise. The data were analyzed with the aid of Clampfit 10.2 software (Molecular Devices) and custom-written program for R (Windows).

### *In Vitro* Studies

#### Binding/Functional Studies

Binding/functional studies were performed commercially in Cerep Laboratories (Poitiers, France) using testing procedures described elsewhere. The general information is listed in Table 4.

#### Electrophysiology

The influence of AS-1 (at a concentration of 100 μM) on fast voltage-gated sodium channels was determined in prefrontal cortex pyramidal neurons. Maximal currents were evoked using rectangular voltage steps from the holding potential of − 65 mV. Control recordings were conducted for 2 min, and the investigated compounds were applied for 3 min. Currents were normalized to control currents. The experimental procedures used in this study adhered to the institutional and international guidelines on the ethical use of animals. Three-week-old rats were decapitated under ethyl chloride anesthesia, and their brains were removed. The methodology of slice preparation and slice preincubation was the same as in our previous study [46]. Parts of the slices containing the prefrontal cortex were mechanically and enzymatically dispersed, exactly the same as in our previous study [46]. Prefrontal cortex pyramidal neurons were visualized using a Nikon inverted microscope. Pipette solution contained the following (in mM): CsF (110), NaCl (7), EGTA (3), HEPES-Cl (10), MgCl_2_ (2), and Na_2_ATP (4) at pH 7.4 and osmolarity of 290 mOsm. The extracellular solution contained the following components (in mM): NaCl (30), choline chloride (90), TEA-Cl (30), CaCl_2_ (2), MgCl_2_ (2), glucose (15), HEPES (10), LaCl_3_ (0.001), and CdCl_2_ (0.4) at pH 7.4. Currents were recorded using an Axopatch 1D amplifier and analyzed with pClamp software (Molecular Devices). Patch pipettes had resistances between 4 and 5 MΩ. After gigaseal formation, the electrode capacitance was compensated. The patch membrane was ruptured by suction, and the membrane capacitance was compensated. The access resistance was between 5 and 7 MΩ. A series resistance compensation of 80% was applied. The sodium currents were leak subtracted. Recordings were performed at room temperature. Voltage-gated potassium currents were not recorded because they were blocked by TEA-Cl in the extracellular solution. Moreover, there were no potassium ions in the intracellular and extracellular solution. Voltage-gated calcium currents were blocked by cadmium and lanthanum ions in the extracellular solution. Physiological holding potential (− 65 mV) was used throughout the study. The AS-1 compound was applied to the whole bath.

#### *In Vitro* ADME-Tox Studies

The ADME-Tox parameters including permeability, metabolic stability, DDIs, and hepatotoxicity were carried out as described previously [9–11, 13, 47]. The ability of AS-1 to passively penetrate through the biological membranes was estimated by Gentest Pre-coated PAMPA Plate System (Corning, Tewksbury, MA) and expressed as the permeability coefficient *Pe*. The human metabolism of the compound AS-1 was studied using human liver microsomes (HLMs) provided by Sigma-Aldrich. The potential DDIs were predicted by luminescent CYP3A4, CYP2D6, and CYP2C9 P450-Glo assays (Promega, Madison, WI). The respective strong CYP’s inhibitors, ketoconazole (KE, half maximal inhibitory concentration (IC_50_) = 0.14 μM), quinidine (QD, IC_50_ = 0.01 μM), and sulfaphenazole (SE, IC_50_ = 0.08 μM), were used as the references. The hepatic safety of AS-1 was estimated here using hepatoma HepG2 cell growth. The cells were seeded in a 96-well plate and incubated in the presence of AS-1 at the concentration range 0.1–100 μM. One micromolar of cytostatic drug doxorubicin (DX) and 10 μM of mitochondrial toxin carbonyl cyanide 3-chlorophenylhydrazone (CCCP) were used as the references.

### Statistical Analysis

Most of the results were analyzed using one-way analysis of variance (ANOVA) with Bonferroni’s post hoc test. The EEG results were analyzed using two-way ANOVA, with Bonferroni’s post hoc test, with the following factors: (1) for pretreatment, vehicle or AS-1, and (2) for treatment, vehicle or PTZ. Statistical evaluation of isobolographic interactions was performed by the use of Student’s *t* test to detect the differences between the experimentally derived (ED_50 mix_) and theoretical additive (ED_50 add_) values, according to Tallarida [30]. Qualitative variables from the chimney test were compared by using Fisher’s exact probability test. Median retention times obtained in the passive avoidance task were statistically evaluated using the Kruskal–Wallis nonparametric ANOVA. Data from electrophysiological studies were compared with Student’s *t* test.

Differences among values were considered statistically significant if *p* < 0.05. All statistical tests were performed using GraphPad Prism (version 5.0) for Windows (GraphPad Software, San Diego, CA).

## Results

### Anticonvulsant Activity in the 6-Hz (44 mA) Seizure Model

Table 1 summarizes the quantitative data for AS-1 and VPA. In this model of pharmacoresistant seizures, the compound AS-1 revealed relatively potent activity. For comparison, the ED_50_ value for the reference drug VPA was obtained in the same experimental conditions.

**Table 1:** The quantitative data for AS-1 and VPA in the 6-Hz (44 mA) test in mice

Compound	TPE (h)	ED_50_ 6-Hz (44 mA) (mg/kg)^a^	TD_50_ (mg/kg)^b^	PI (TD_50_/ED_50_)^c^
AS-1	0.5	75.41 (63.60–89.42)	347.6 (132.7–288.6)^d^	4.6
VPA	0.5	183.1 (143.5–233.7)	430.7 (407.9–454.9)^d^	2.3

AS-1 and VPA were injected i.p., 30 min before the tests. Values in parentheses are 95% confidence intervals determined by probit analysis

TPE = time to peak effect

^a^ED_50_ (6-Hz, psychomotor seizure test, 44 mA)

^b^TD_50_ (NT, acute neurological toxicity determined in the rotarod test)

^c^Protective index (TD_50_/ED_50_)

^d^Data from Kamiński et al. [10]

### Effect of Repeated Treatment with AS-1 on Seizure Severity in the PTZ-Induced Kindling in Mice

**Fig. 2:**
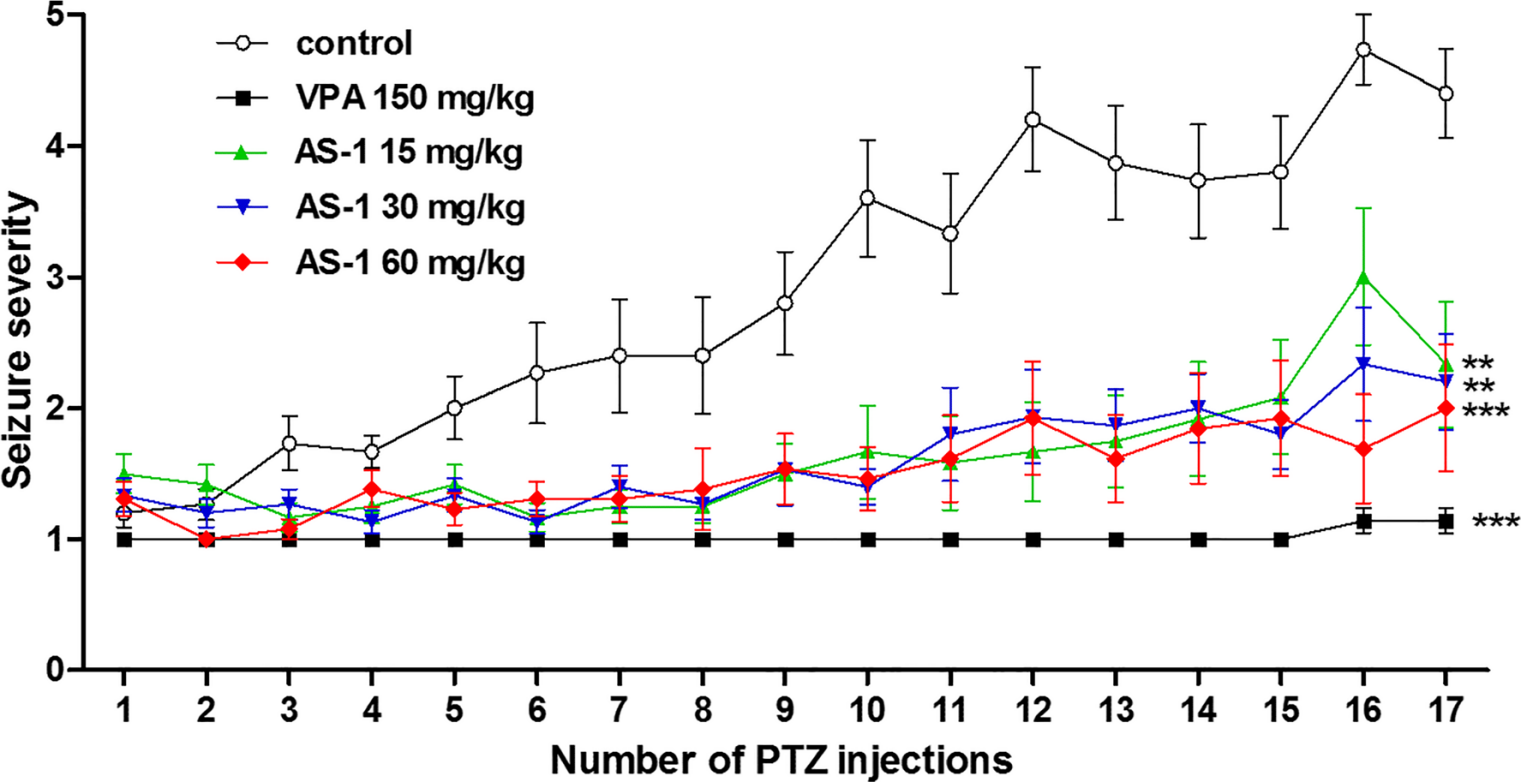
Effect of AS-1 on kindling development in mice. Kindling was induced by repeated injections of PTZ (40 mg/kg, i.p.) every 48 h for a total of 17 injections. AS-1 and VPA were injected i.p. once daily for 33 days. Experimental groups consisted of 12–15 animals. Data are expressed as means ± SEM. Statistical analysis: one-way ANOVA followed by Bonferroni’s post hoc test. **p < 0.01, ***p < 0.001, as compared to the kindled control group

Figure 2 shows the effect of repeated treatment with AS-1 on the PTZ kindling progression in mice (one-way ANOVA: *F*(4,64) = 9.66, *p* < 0.0001, on the 33rd day). The repeated administration of PTZ at a subconvulsive dose (40 mg/kg) on every alternate day for a total of 17 injections caused a gradual increase in the mean seizure severity score in the control group (from 1.2 ± 0.1 after the first PTZ injection to 4.4 ± 0.3 after the last PTZ injection). Repeated treatment (a total of 33 injections) with AS-1 at doses of 15 mg/kg, 30 mg/kg, and 60 mg/kg significantly suppressed kindling development (*p* < 0.01 for AS-1 at 15 mg/kg and 30 mg/kg and *p* < 0.001 for AS-1 at 60 mg/kg *vs* the PTZ-kindled control group). Likewise, VPA (positive control) at 150 mg/kg suppressed kindling progression (*p* < 0.001 *vs* the PTZ-kindled control group).

### Effect of Repeated Treatment with AS-1 on Locomotor Activity in PTZ-Kindled Mice

No significant changes in spontaneous locomotor activity in the PTZ-kindled control group (as compared to the non-kindled control group) were observed (data not shown). Repeated injection of AS-1 (15–60 mg/kg) or VPA (150 mg/kg) did not affect locomotor activity in the kindled mice as well (one-way ANOVA: *F*(5,77) = 1.06, *p* = 0.391).

### Effect of Repeated Treatment with AS-1 in the Elevated Plus Maze Test in PTZ-Kindled Mice

No significant effect of PTZ kindling on animal performance in the elevated plus maze test was observed (Fig. S1). Moreover, repeated injection of AS-1 (15–60 mg/kg) or VPA (150 mg/kg) did not produce any significant changes in both the percentage of open arm entries and the percentage of time spent in open arm entries in kindled animals (one-way ANOVA: *F*(5,77) = 1.27, *p* = 0.286, for the percentage of open arm entries, and *F*(5,76) = 2.90, *p* = 0.019, for the percentage of the time spent in the open arm entries).

### Effect of Repeated Treatment with AS-1 on PTZ Kindling–Induced Depression in Mice

Repeated PTZ injection on every alternate day produced depressive-like behavior in mice, which was demonstrated by an increase in the total immobility duration in the forced swim test (*p* < 0.001 *vs* the non-kindled control group). VPA injected repeatedly at a dose of 150 mg/kg slightly attenuated the PTZ-induced behavioral despair and significantly decreased the immobility time in kindled animals (*p* < 0.05 as compared to the kindled control group). However, repeated injection of AS-1 (15–60 mg/kg) did not produce any significant changes in the total immobility duration in comparison to the PTZ-kindled control group (one-way ANOVA: *F*(5,75) = 15.14, *p* < 0.0001; Fig. S2).

### Effect of Repeated Treatment with AS-1 on the Seizure Threshold in the i.v. PTZ Test in Kindled Mice

**Fig. 3:**
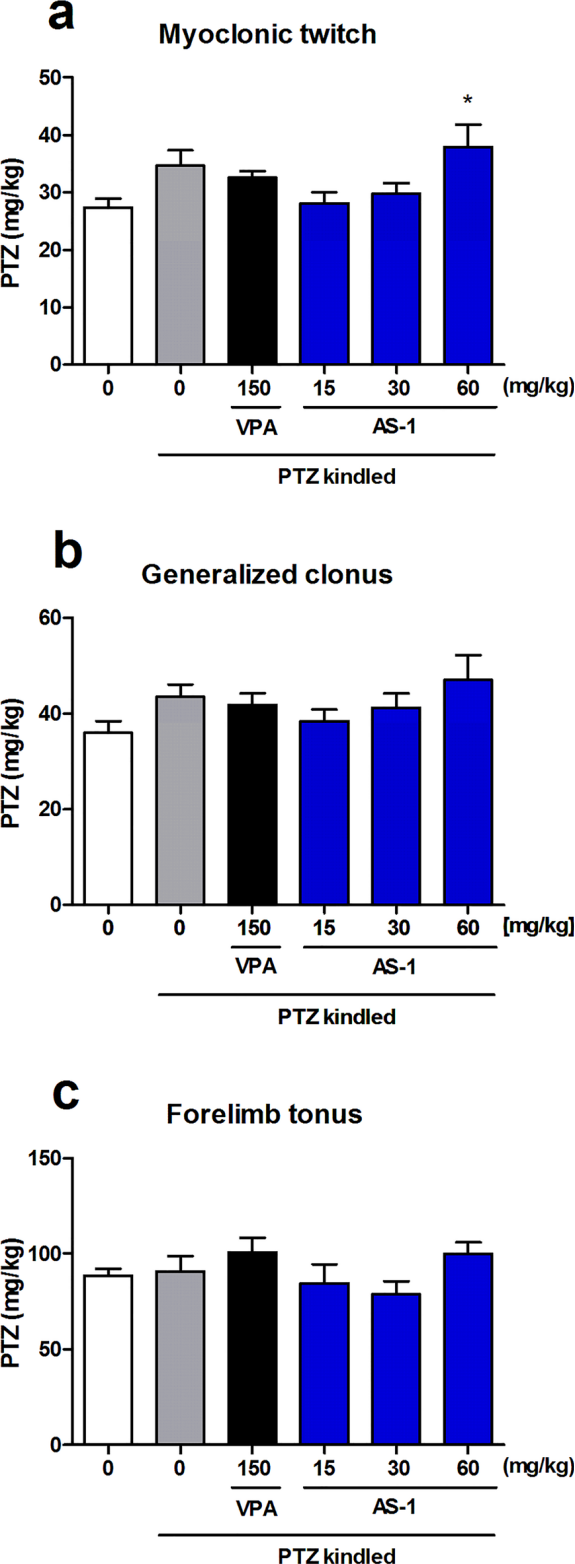
Effect of repeated treatment with AS-1 on the threshold for the first myoclonic twitch (panel **a**), generalized clonus (panel **b**), and forelimb tonus (panel **c**) in the i.v. PTZ seizure test in kindled mice. PTZ (40 mg/kg, i.p.) was injected every 48 h for a total of 17 injections. AS-1 and VPA were injected i.p. once daily for 33 days. Experimental groups consisted of 8–15 animals. Data are expressed as means + SEM. Statistical analysis: one-way ANOVA followed by Bonferroni’s post hoc test. *p < 0.05, as compared to the non-kindled control group

The effect of repeated AS-1 injection on the seizure thresholds in the i.v. PTZ test in kindled mice is shown in Fig. 3 a–c (one-way ANOVA: *F*(5,63) = 2.86, *p* = 0.022, for myoclonic twitch; *F*(5,66) = 1.58, *p* = 0.178, for generalized clonus; and *F*(5,59) = 1.43, *p* = 0.228, for forelimb tonus). PTZ-induced kindling had no effect on thresholds for the first myoclonic twitch, generalized clonic seizure, and forelimb tonus. Repeated injection of AS-1 at the highest dose tested (60 mg/kg) significantly increased the seizure threshold for the first myoclonic twitch (*p* < 0.05 as compared to the non-kindled control group). No changes in the thresholds for generalized clonus or forelimb tonus after repeated pretreatment with AS-1 (15–60 mg/kg) were observed.

### Effect of Acute Administration of AS-1 on the Seizure Threshold in the i.v. PTZ Test in Naïve Mice

**Fig. 4:**
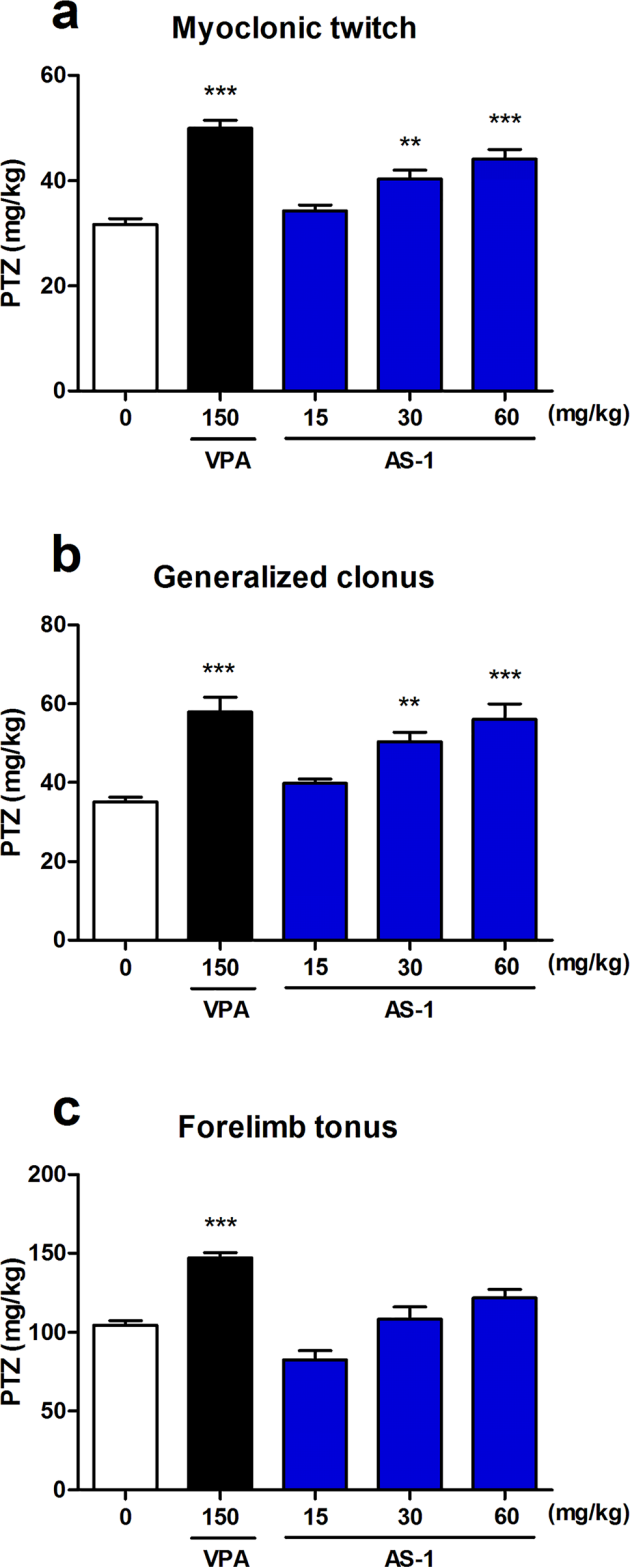
Effect of acute administration of AS-1 on the threshold for the onset of first myoclonic twitch (panel **a**), generalized clonus (panel **b**), and forelimb tonus (panel **c**) in the i.v. PTZ seizure threshold test in naïve mice. AS-1 and VPA were injected i.p. 30 min and 15 min before the test, respectively. Experimental groups consisted of 10–14 animals. Data are expressed as means + SEM. Statistical analysis: one-way ANOVA followed by Bonferroni’s post hoc test: **p < 0.01, ***p < 0.001, as compared to the control group

The effect of AS-1 administered acutely on the seizure thresholds in the i.v. PTZ test in naïve mice is shown in Fig. 4 a–c (one-way ANOVA: *F*(4,57) = 23.11, *p* < 0.0001, for myoclonic twitch; *F*(4,57) = 11.90, *p* < 0.0001, for generalized clonus; and *F*(4,53) = 15.62, *p* < 0.0001, for forelimb tonus). AS-1 injected at a dose of 15 mg/kg did not affect significantly the thresholds for the first myoclonic twitch and generalized clonus. However, AS-1 administered at doses of 30 mg/kg and 60 mg/kg raised the thresholds for both the first myoclonic twitch (*p* < 0.01 and *p* < 0.001, respectively) and generalized clonic seizure (*p* < 0.01 and *p* < 0.001, respectively). Acute administration of AS-1 (15–60 mg/kg) did not affect the susceptibility of mice to the PTZ-induced forelimb extension.

### Log-Probit Dose–Response Relationship Line Analysis for AS-1 and VPA Against PTZ-Induced Clonic Seizures in Mice

**Fig. 5:**
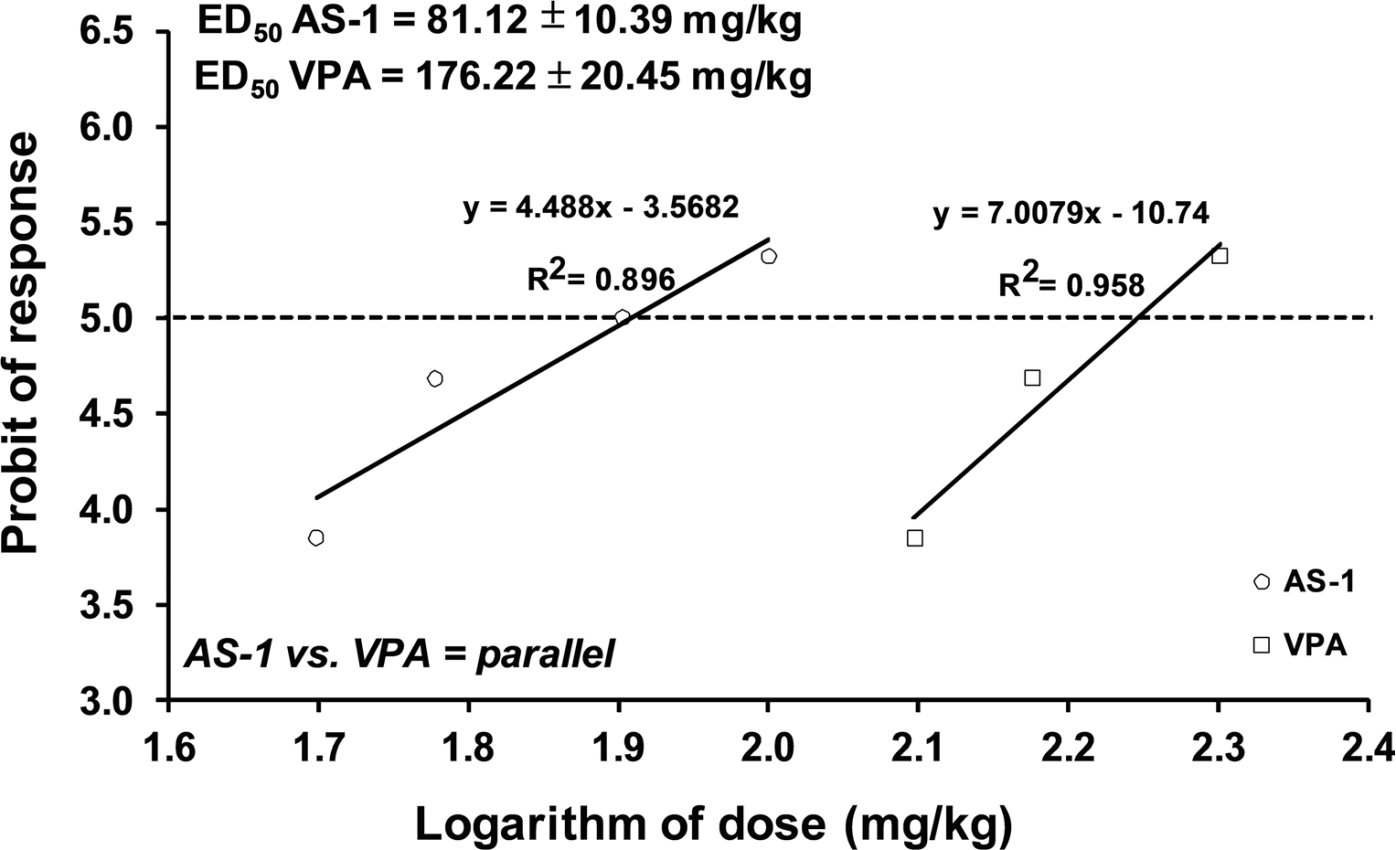
Log-probit dose–response relationship lines for AS-1 and VPA administered alone in the mouse PTZ-induced clonic seizure model. Doses of AS-1 and VPA were transformed in logarithms, whereas the protective effects offered by the drugs administered alone against PTZ-induced seizures were transformed in probits of response according to Litchfield and Wilcoxon [25]. The equations of dose–response relationship lines for AS-1 and VPA are presented on the graph, where *y* is the probit of response, *x* is the logarithm to the base 10 of drug doses, and *R*^2^ is the coefficient of determination. The test for parallelism of two dose–response relationship lines revealed that both dose–response relationship lines were parallel

In the PTZ test, AS-1 was administered separately at the increasing doses from 50 to 100 mg/kg. Subsequently, log-probit transformation of the data allowed the determination of the equation of dose–response relationship for AS-1 administered alone, which was *y* = 4.488*x* − 3.5682 [*R*^2^ = 0.8962] (Fig. 5). The estimated ED_50_ value for AS-1 was 81.12 ± 10.39 mg/kg. With respect to VPA, the drug was given at doses of 125 mg/kg, 150 mg/kg, and 200 mg/kg, and the protection (in %) against PTZ-induced clonic seizures was 12.5%, 37.5%, and 62.5%, respectively. The equation of dose–response relationship for VPA was *y* = 7.0079*x* × 10.74 [*R*^2^ = 0.9586]. The ED_50_ for VPA was 176.22 ± 20.45 mg/kg (Fig. 5).

### Isobolographic Characteristic of the Interaction Between AS-1 and VPA in PTZ-Induced Seizures in Mice

**Fig. 6:**
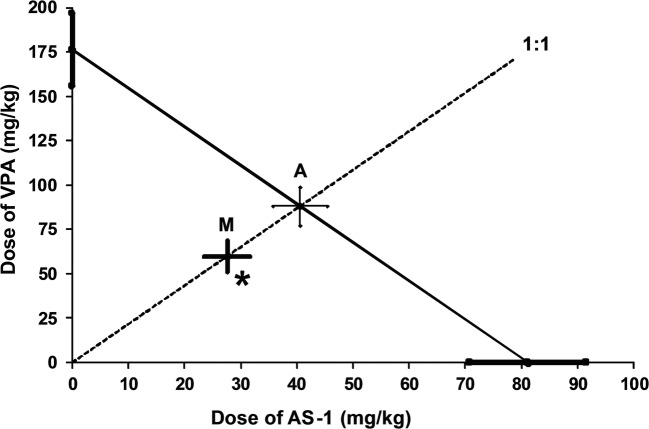
Isobologram showing the interaction between AS-1 and VPA against PTZ-induced clonic seizures in mice. The ED_50_ value for AS-1 is plotted graphically on the *x*-axis, whereas the ED_50_ value of VPA is plotted on the *y*-axis. The solid lines on the *x*- and *y*-axes represent the 95% confidence limits (CLs) for the drugs administered alone. The straight line connecting these two ED_50_ values on the graph represents the theoretical line of additivity for a continuum of 1:1 fixed dose ratios. A = ED_50 add_; M = ED_50 mix_. All 95% CLs of A and M values are presented horizontally and vertically in the shape of a cross. The experimental ED_50 mix_ values of the mixture of AS-1 + VPA (M) for the fixed ratio of 1:1 are placed below the theoretical line of additivity, indicating the supra-additive (synergistic) interactions; *p < 0.05

The isobolographic analysis showed that a combination of AS-1 and VPA at the fixed ratio of 1:1 displayed supra-additive (synergistic) interactions against PTZ-induced seizures in mice, because the experimentally denoted ED_50 mix_ (87.48 mg/kg) considerably differed from the theoretically calculated ED_50 add_ (128.67 mg/kg) (*p* < 0.05) (Table 2, Fig. 6).

**Table 2:** Isobolographic analysis of the interaction between AS-1 and VPA at the fixed-ratio combination of 1:1 in the s.c. PTZ test in mice

Combination	ED_50 add_	*n*_add_	ED_50 exp_	*n*_exp_	Alpha	Interaction
AS-1 + VPA	128.67 ± 15.42	44	87.48 ± 12.75*	24	0.68	synergy

AS-1 and VPA were injected i.p., 30 min before the test. Data are presented as ED_50_ values ± SEM. The clonic phase of PTZ-induced seizures was produced by the s.c. injection of PTZ (100 mg/kg). The ED_50_ values were either experimentally determined from the mixture of AS-1 and VPA (ED_50 mix_) or theoretically calculated from the equation of additivity (ED_50 add_); *n* is the total number of animals used at those doses whose expected anticonvulsant effects were ranged between 4 and 6 probits, denoted for the experimental mixture of drugs (*n*_mix_) and theoretically calculated (*n*_add_) from the equation of additivity; alpha is the interaction index (ED_50 mix_/ED_50 add_)

*p < 0.05, *versus* the theoretically additive ED_50 add_ value (unpaired Student’s *t* test with Welch correction)

### Effect of AS-1 Administered Alone and in Combinations with VPA on Long-Term Memory, Motor Coordination, and Skeletal Muscular Strength in Mice

When AS-1 was co-administered with VPA at the fixed ratio of 1:1, the motor coordination in mice was unaffected. Furthermore, the studied combinations did not impair long-term memory as determined in the passive avoidance task, with the median retention time being 180 s. Similarly, this combination had no effect on muscular strength, as assessed by the grip strength test (Table 3). Moreover, AS-1 and VPA administered alone at doses of 81.12 mg/kg and 176.22 mg/kg, respectively, being their ED_50_ values from the PTZ test, did not significantly affect long-term memory, muscular strength, and motor performance in mice (Table 3).

**Table 3:** Effects of AS-1, VPA, and their combinations on long-term memory, skeletal muscular strength, and motor coordination in mice

Treatment (mg/kg)	Retention time (s)^a^	Muscular strength (g)^b^	Motor coordination (%)^c^
Control	180 (156; 180)	102.1 ± 2.6	0
AS-1 (81.12) + vehicle	180 (146; 180)	99.9 ± 2.2	0
VPA (176.22) + vehicle	180 (180; 180)	104.7 ± 2.8	12.5
AS-1 (27.6) + VPA (59.9)	180 (160; 180)	104.2 ± 4.7	0

Results are presented as follows: (a) median retention times (in seconds; with the 25th and 75th percentiles in parentheses) from the passive avoidance task, assessing long-term memory in mice; (b) mean grip strengths (in grams ± SEM) from the grip strength test, assessing muscular strength in mice; and (c) percentage of animals showing motor coordination impairment in the chimney test in mice. AS-1 and VPA were injected i.p., 30 min before the tests. Each experimental group consisted of 8 animals. Statistical analysis of data from the passive avoidance task was performed with the Kruskal–Wallis nonparametric ANOVA test followed by Dunn’s post hoc test, whereas those from the grip strength test were analyzed with one-way ANOVA followed by Bonferroni’s post hoc test. Fisher’s exact probability test was used to analyze the results from the chimney test

### Electrographic Discharge Assessment in Zebrafish

**Fig. 7:**
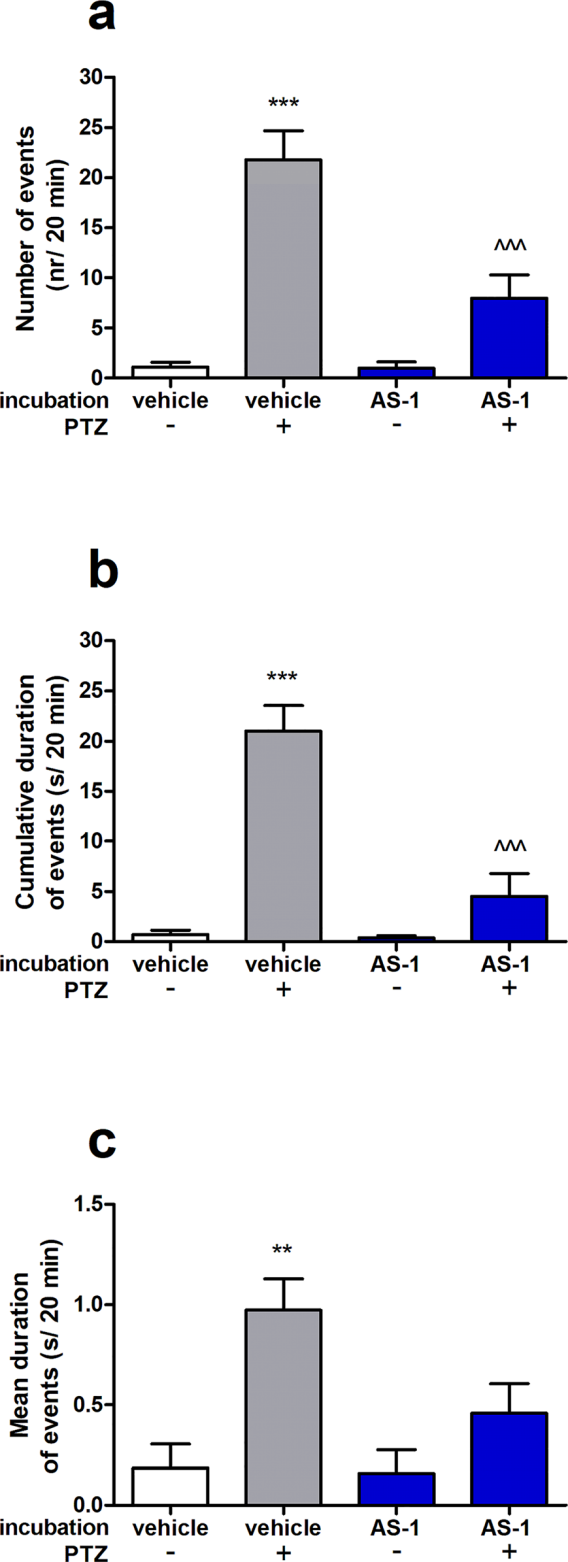
Electrophysiological recordings from the optic tectum of larvae pre-exposed to vehicle or AS-1. Results are presented as the number of epileptiform events during 20 min (**a**), the cumulative duration of epileptiform events during 20 min (**b**), and the mean duration of the event (**c**). Experimental groups consisted of 5–12 animals. Statistical analysis: two-way ANOVA followed by Bonferroni’s post hoc test. **p < 0.01, ***p < 0.001, as compared to the vehicle + vehicle group; ^^^*p* < 0.001, as compared to the vehicle + PTZ group

The EEG recordings from the optic tectum of zebrafish larvae exposed to acute PTZ (20 mM) were performed to confirm the anticonvulsant potential of AS-1 (5 mM). Exposure to AS-1 significantly reduced both numbers (Fig. 7a; two-way ANOVA, pretreatment: *F*(1,32) = 8.02, *p* < 0.01; treatment: *F*(1,32) = 31.99, *p* < 0.001; pretreatment × treatment interaction: *F*(1,32) = 7.79, *p* < 0.01; *n* = 5–12/group) and total duration (Fig. 7b; two-way ANOVA, pretreatment: *F*(1,32) = 13.58, *p* < 0.001; treatment: *F*(1,32) = 28.98, *p* < 0.001; pretreatment × treatment interaction: *F*(1,32) = 12.55, *p* < 0.001; *n* = 5–12/group) of epileptiform discharges in the zebrafish brain in comparison to the control group which was incubated in the vehicle. Additionally, AS-1 slightly decreased the mean duration of events, but results did not reach statistical significance (Fig. 7c; two-way ANOVA, pretreatment: *F*(1,32) = 5.58, *p* > 0.05; treatment: *F*(1,32) = 22.39, *p* < 0.01; pretreatment × treatment interaction: *F*(1,32) = 4.52, *p* > 0.05; *n* = 5–12/group).

### Binding/Functional Studies

In order to identify the plausible pharmacodynamics of the compound AS-1, we performed binding/functional assays for several molecular targets for anticonvulsants. Moreover, bearing in mind cardiac safety, the influence of AS-1 on potassium channel (hERG) was determined as well (Table 4).

**Table 4:** *In vitro* binding/functional assays for AS-1

	Source		Ref.
		% inhibition of control-specific binding (concentration in μM)	
Binding studies			
Na^+^ channel (site 2)^a^	Rat cerebral cortex	15.9 (100) 56.0 (500)*	[35]
L-type Ca^2+^ (dihydropyridine site, antagonist radioligand)^a^	Rat cerebral cortex	− 14.8 (100)	[36]
L-type Ca^2+^ (verapamil site, antagonist radioligand)^a^	Rat cerebral cortex	15.0 (100) 45.0 (500)	[37]
L-type Ca^2+^ (diltiazem site, antagonist radioligand)	Rat cerebral cortex	10.2 (100)	[38]
N-type Ca^2+^ (antagonist radioligand)^a^	Rat cerebral cortex	0.2 (100)	[39]
NMDA (antagonist radioligand)^a^	Rat cerebral cortex	15.0 (100) 35.0 (200)	[40]
GABA_A1_ (α_1_β_2_γ_2_) (agonist radioligand)^a^	Rat cerebral cortex	− 18.3 (100)	[41]
GABA transporter (antagonist radioligand)	Rat cerebral cortex	2.4 (100)	[42]
Potassium channel (hERG)	Human recombinant (HEK-293 cells)	0.8 (100)	[43]
		% inhibition of control agonist/antagonist response (concentration in μM)	
Functional studies			
TRPV1 (VR1) (*h*) (antagonist effect)	Human recombinant (CHO cells)	4.3 (100)	[44]
CB1 (*h*) (agonist effect)	Human recombinant (CHO cells)	− 6.8 (100)	[45]
CB1 (*h*) (antagonist effect)	Human recombinant (CHO cells)	5.6 (100)	[45]

^a^Data from Rapacz et al. [12]

*Results showing an inhibition higher than 50% are considered to represent significant effects of the test compounds, results showing an inhibition between 25 and 50% are indicative of weak effect, and results showing an inhibition lower than 25% are not considered significant and mostly attributable to the variability of the signal around the control level

### Electrophysiology

**Fig. 8:**
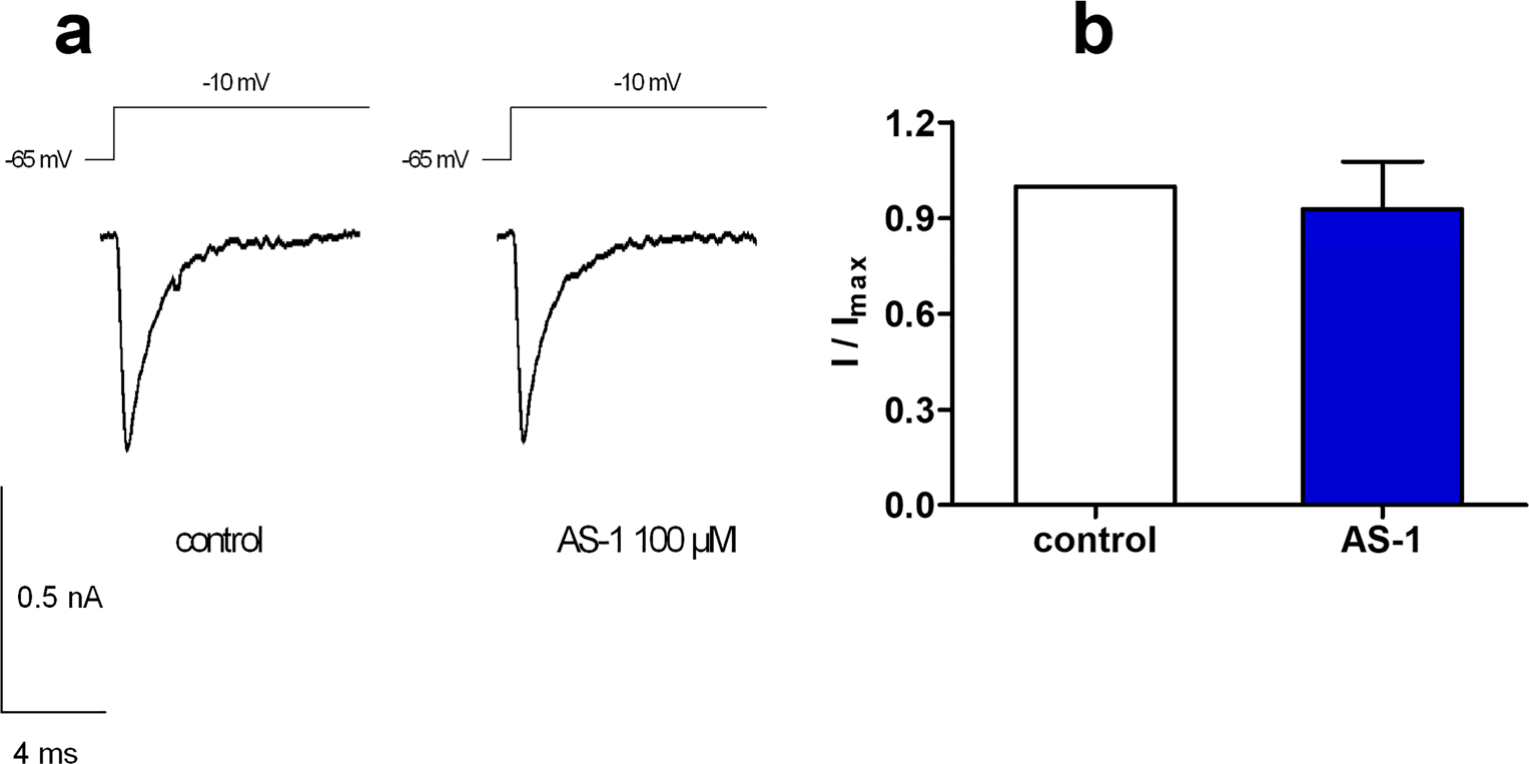
The influence of AS-1 on fast voltage-gated sodium current in prefrontal cortex pyramidal neurons. (**a**) Example recordings of maximal voltage-gated sodium currents in the control and in the presence of AS-1. Currents were evoked by rectangular voltage steps to − 10 mV, as shown above the current traces. (**b**) Averaged normalized maximal sodium current amplitudes in the control and in the presence of AS-1. Statistical analysis: Student’s *t* test

AS-1 did not influence the maximal amplitude of sodium currents (1.0 in control and 0.93 ± 0.06 after the addition of the tested compound, *n* = 6, *p* > 0.05). Example recordings of sodium currents and averaged results are shown in Fig. 8 a and b, respectively.

### *In Vitro* ADME-Tox Studies

The references used in the parallel artificial membrane permeability assay (PAMPA) test included low-permeable norfloxacin (NFX, *Pe* = 0.56 ± 0.01 × 10^−6^ cm/s) and well-permeable caffeine (CFN, *Pe* = 15.1 ± 0.04 × 10^−6^ cm/s). AS-1 showed permeability with calculated *Pe* = 9.8 ± 2.0 × 10^−6^ cm/s (Table 5).

**Table 5:** *In vitro* ADME-Tox parameters of AS-1

Assay	Result
*Pe* (10^−6^ cm/s ± SD)	9.8 ± 2.0^a^
Phase I metabolism in human	No metabolites found
CYP3A4 activity*	96.9 ± 0.4^a^
CYP2D6 activity*	104.4 ± 0.5^a^
CYP2C9 activity*	67.0 ± 3.6^a^
HepG2 viability**	82.3 ± 6.5^a^

*Percentage of control ± SD at 10 μM

**Percentage of control ± SD at 100 μM

^a^Tested in at least 3 repetitions

**Fig. 9:**
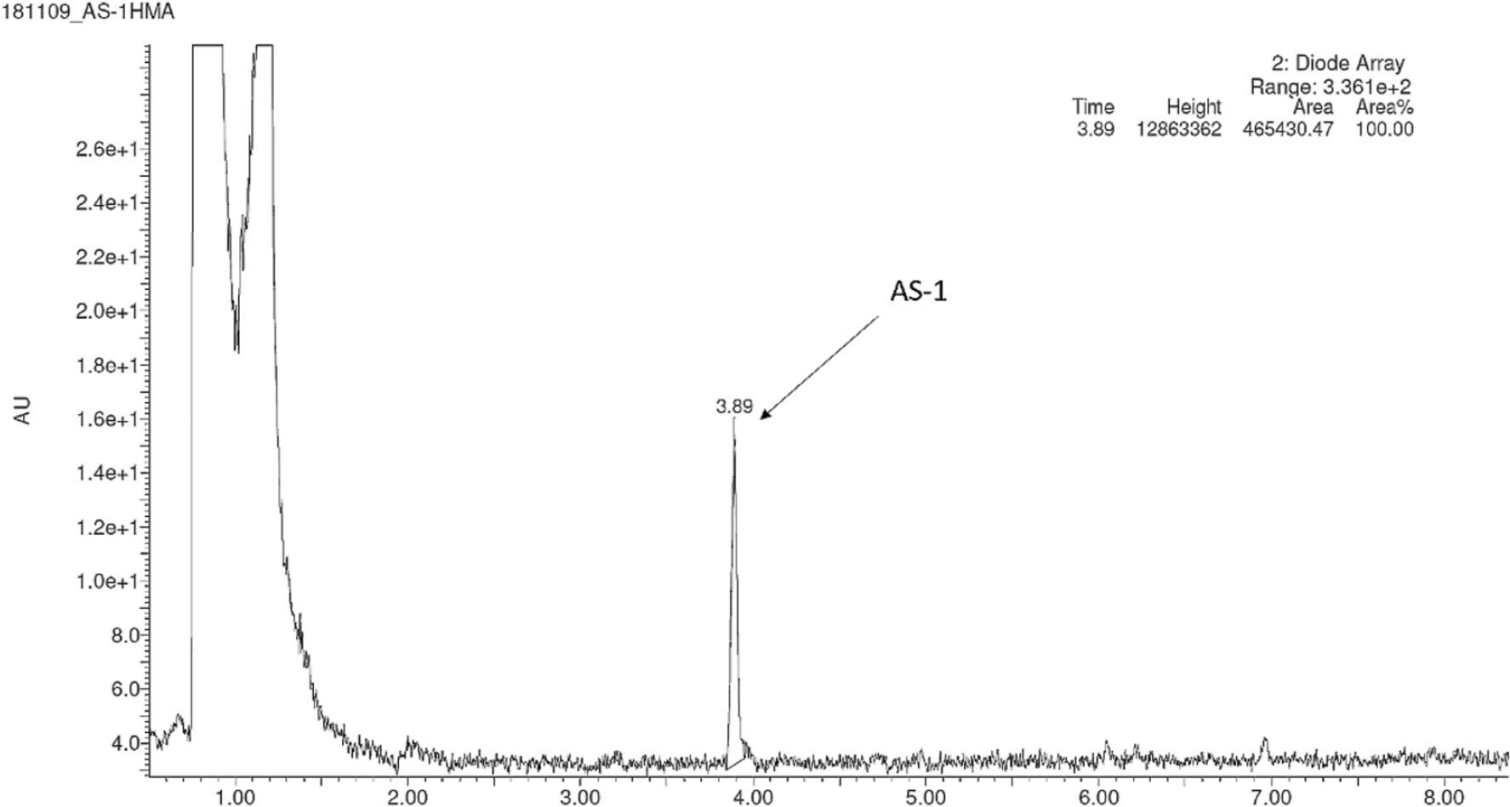
UPLC spectra of the reaction mixture after the 120-min incubation of AS-1 with HLMs

The UPLC analysis performed after 120 min of AS-1 incubation with HLMs showed no presence of metabolites (Fig. 9).

**Fig. 10:**
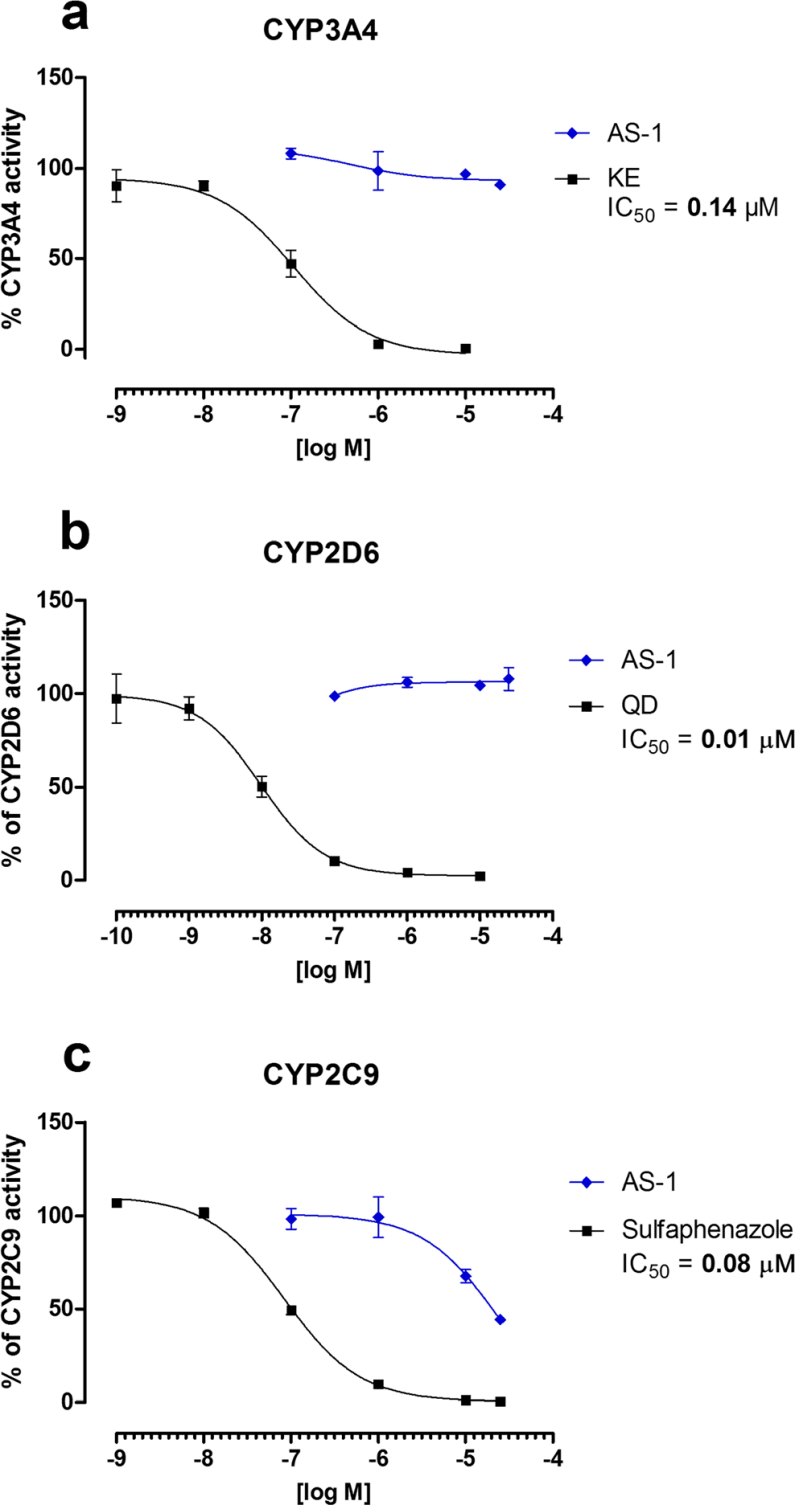
The effect of the respective inhibitor and compound AS-1 on the CYP3A4 (**a**), CYP2D6 (**b**), and CYP2C9 (**c**) activity

No significant effect of AS-1 on CYP3A4 and CYP2D6 activity was observed (Fig. 10a, b), whereas moderate CYP2C9 inhibition was determined at the highest used doses of 10 μM and 25 μM (Fig. 10c). For detail, see also data in Table 5.

**Fig. 11:**
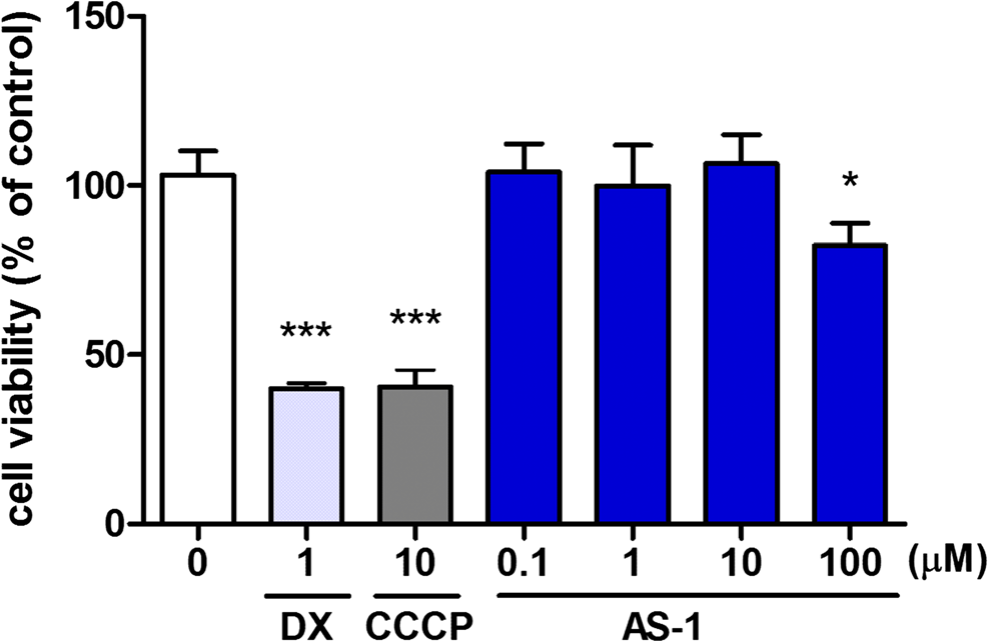
The effects of AS-1 and reference toxins (DX = doxorubicin; CCCP = carbonyl cyanide 3-chlorophenylhydrazone) on the hepatoma HepG2 cell line viability. Statistical analysis: one-way ANOVA followed by Bonferroni’s post hoc test. *p < 0.05, ***p < 0.001

The slight but statistically significant effect of AS-1 (*p* < 0.05) on hepatoma HepG2 cells’ viability was observed only at the highest concentration used of 100 μM. The reference toxins DX and CCCP decreased cells’ viability to ~ 40% of control at 1 μM and 10 μM, respectively (Fig. 11, Table 5).

## Discussion

In our previous study, AS-1 has been identified as a potent broad-spectrum anticonvulsant agent. It produced a clear-cut protective effect in three acute seizure tests in mice (i.e., MES, s.c. PTZ, and 6-Hz at 32 mA) [10, 12]. With the aim of completing the anticonvulsant characterization of AS-1 in acute seizure models in the current studies, we applied the 6-Hz test at 44 mA. The 6-Hz model with 44 mA stimulus intensity has been described as a useful tool in the identification of novel compounds with potential efficacy against pharmacoresistant seizures [17, 48]. In the previous paper, we demonstrated that AS-1 protected mice against psychomotor seizures in the 6-Hz test with 32 mA stimulus intensity (a model of partial seizures) with an effective dose value of 24.66 mg/kg [12]. In the present study, the ED_50_ value was 75.41 mg/kg. It should be stressed that in some cases, compounds may effectively block seizures at a lower stimulus intensity of 32 mA but not at 44 mA. For LEV, the ED_50_ value is at least 50 times higher at the 44 mA stimulus intensity *versus* the 32 mA [17]. Furthermore, as shown in Table 1, AS-1 revealed distinctly more potent protection in the 6-Hz (44 mA) test and simultaneously offered better safety margin in the rotarod performance test compared to VPA. These results may indicate the potential utility of AS-1 in the treatment of refractory epilepsy in humans.

In the subsequent studies, we aimed to investigate the antiseizure effect of AS-1 in the PTZ-induced kindling model in mice. The obtained results showed that the 33-day treatment with AS-1 (at doses of 15 mg/kg, 30 mg/kg, and 60 mg/kg) decreased the seizure severity score and thereby suppressed kindling progression, which suggests that the tested compound may possess the ability to prevent epilepsy development. However, the observed effect could be related more to the acute anticonvulsant activity of AS-1 rather than to its possible antiepileptogenic-like properties. A different experimental approach is required in order to ascertain whether AS-1 can suppress epileptogenesis.

PTZ is recognized as a noncompetitive GABA_A_ receptor antagonist. It binds to the picrotoxin-sensitive site of the GABA_A_ receptor complex and inhibits the GABA-evoked chloride current [49]. Therefore, drugs effective in PTZ-induced seizure models are generally considered to act via GABAergic mechanisms. Kindling induced by PTZ was shown to produce not only changes in the GABA-mediated neurotransmission but also various alterations in glutamate excitatory neurotransmission, mediated by both NMDA and AMPA receptors, as well as some morphological changes in the hippocampus, glucose hypometabolism, or antioxidant defense systems [50, 51]. Noteworthy, a crucial role of synaptic vesicle glycoprotein 2A (SV2A) in kindling development has been also postulated [52]. Hence, several different mechanisms could be responsible for the antiepileptogenic-like effects of AS-1 in the PTZ-induced kindling model. In our previous report, *in vitro* radioligand binding studies revealed that AS-1 exerted moderate affinity towards NMDA receptors (antagonist radioligand) but it did not bind effectively to the GABA_A1_ receptor α1, β2, and γ2 subunits [12]. Inhibition of the GABAergic neurotransmission following PTZ administration leads to glutamatergic neuronal excitation, and possible antagonistic interactions with NMDA receptors could be, at least in part, responsible for the antiseizure properties of AS-1 in the PTZ kindling model. Nevertheless, this is a suggestion only and further detailed studies are required to evaluate the influence of AS-1 on glutamatergic neurotransmission. Moreover, the tested compound (at high concentration) was also shown to have moderate binding affinity for the L-type Ca^2+^ channels (verapamil site) [12]. Although the L-type Ca^2+^ channels are not the main target for antiepileptic therapy, they may also play a role in epileptogenesis [53].

It is widely known that the prevalence of depressive and anxiety disorders is much higher in epileptic patients than in general population. To evaluate the effect of AS-1 on these two epilepsy-related comorbidities, the animals were subjected to the elevated plus maze test and the forced swim test 24 h after the last PTZ injection. No significant changes in the anxiety-related response in the elevated plus maze test were reported. However, we found that PTZ-induced kindling led to the depressive-like behavior in mice, which is in agreement with many previous reports [54–56]. Repeated injection of AS-1 did not alleviate the kindling-induced behavioral despair in mice. The animals’ performance in the elevated plus maze test and the forced swim test was not affected by changes in the spontaneous locomotor activity in mice. It is worth mentioning that in our former study, another hybrid compound—KA-11 (1-(1-oxo-1-(4-(3-(trifluoromethyl)phenyl)piperazin-1-yl)propan-2-yl)pyrrolidine-2,5-dione)—prevented the depressive-like behavior caused by the PTZ-induced kindling in mice [13].

In the next experiment, the effect of repeated AS-1 injection on the seizure threshold following PTZ kindling was investigated. The timed i.v. PTZ seizure test was used as one of the most sensitive methods for assessing seizure thresholds in rodents. The test was performed 48 h after the last PTZ injection in the kindling model. A slight increase in the seizure threshold for myoclonic seizures was observed only in animals treated with AS-1 at the highest dose tested, i.e., 60 mg/kg. No changes in the seizure threshold for generalized clonic seizure and tonic seizure were noted. The obtained results suggest that AS-1, in contrast to compound KA-11, should not induce withdrawal hyperexcitability. In our previous study, KA-11 produced the withdrawal-induced reduction in seizure susceptibility [13]. The acute effects of AS-1 on seizure threshold in the i.v. PTZ test have not been previously studied. Therefore, we also investigated the influence of AS-1 injected acutely on seizure susceptibility in naïve (i.e., non-kindled) animals. AS-1 raised the threshold for both the first myoclonic twitch and generalized clonus, but it did not increase the threshold for forelimb tonus. Thus, it seems that AS-1 was not able to slow down the progression of seizures from generalized clonus with a loss of righting reflex to forelimb tonic extension. This is quite an unexpected result because AS-1 was shown to protect against tonic seizures in the MES test [10]. PTZ-induced seizures are particularly sensitive to compounds that act by enhancing the GABAergic neurotransmission, whereas the MES test is thought to be useful for discovery of new anticonvulsants that inactivate sodium channels [57]. Therefore, it seems that AS-1 inhibits tonic seizures rather by blocking sodium channels than by GABAergic mechanisms. This issue warrants further investigation because AS-1 did not inhibit voltage-gated sodium currents in rat cortical neurons. It did, however, bind to the neuronal sodium channels (site 2) but only at the very high concentration of 500 μM [12].

As previously mentioned, AEDs enhancing GABA-mediated inhibition in the brain are effective in the suppression of clonic seizures in rodents after the s.c. injection of PTZ [27, 58]. Thus, the PTZ test is considered as a model of myoclonic and absence seizures in epileptic patients [59, 60]. Bearing in mind the antiseizure properties of AS-1 in the PTZ-induced kindling model in mice in the subsequent studies, we investigated its interaction with VPA using the PTZ as proconvulsant agent. Results from isobolographic analysis indicated that the combination of AS-1 and VPA, at a fixed ratio of 1:1, exerted a supra-additive (synergistic) interaction against PTZ-induced clonic seizures in mice. This is probably an outcome of the mechanisms of action of both drugs. One of the mechanisms of action for VPA is GABA levels increasing in the whole brain and enhancing GABAergic activity [61, 62]. We can speculate that AS-1 also acts through the GABA system, so the combination of both VPA and AS-1 results in a statistically significant decrease of drug doses leading to a supra-additive effect. It should be emphasized that AEDs producing a synergistic interaction in isobolography may prove advantageous in the clinical settings mainly due to the reduction of drugs’ doses. Furthermore, results from the behavioral studies showed that AS-1 and VPA administered alone and in combination at a fixed ratio of 1:1 (at doses corresponding to their ED_50_ values from the s.c. PTZ test) did not cause any disturbances in the chimney, passive avoidance, and grip strength tests in mice. The lack of acute adverse effects results from the reduction of AS-1 and VPA doses, which suggests that it would be a safe combination in further more advanced clinical studies.

In our previous paper, we proved the efficacy of AS-1 in different acute seizure tests in mice. To complement our observation, herein, we assessed its activity in larval zebrafish by means of EEG recordings. We found out that the preincubation of larval zebrafish with AS-1 decreased the number and cumulative duration of EEG discharges induced by acute PTZ. This proconvulsant in larval zebrafish induces a number of behavioral changes, starting with burst swimming and culminating in tonic–clonic convulsions, resembling epileptic seizures in mammals [34, 63, 64]. At the EEG level, exposure to PTZ induces frequent, recurrent ictal-like discharges [33, 65]. To date, the zebrafish PTZ assay has been validated and extensively characterized using commercially available AEDs [33, 66], while obtained results have been proved to translate well to rodent models [34, 65, 67, 68].

In our previous study, we did not manage to explain the potential mechanism of action for AS-1 satisfactorily [12]. We noted only a moderate binding to sodium channel (site 2), verapamil site of the L-type Ca^2+^ channel, and NMDA receptor, however only at the very high concentration of 100 μM or 500 μM (for details, see Table 4). Therefore, on the basis of these data, it is hard to hypothesize about clear and certain pharmacodynamics of AS-1. To extend the *in vitro* characterization of AS-1 that could potentially define the plausible mechanism of action, we decided herein to investigate its influence on the L-type Ca^2+^ channel (diltiazem site), GABA transporter, and the newest and currently attractive targets for anticonvulsants such as TRPV1 (receptor potential cation channel vanilloid type 1 ion channel) and cannabinoid CB1 receptors [69, 70] (Table 4). Notably, the interaction between TRPV1 and CB1 receptors is crucial for the pharmacodynamics of cannabidiol, which is currently used in children with pharmacoresistant epilepsy, such as Dravet or Lennox–Gastaut syndromes [69, 71, 72]. As a result, in the binding/functional studies, AS-1 did not interact with the L-type Ca^2+^ channel (diltiazem site), GABA transporter, or TRPV1 and CB1 receptors at a high concentration of 100 μM. Thus, on the basis of the previous and current binding and/or functional assays, the mechanism of action for AS-1 remains undefined. We can only state that the broad-spectrum anticonvulsant activity in the acute and kindling models of epilepsy observed for AS-1 in the preclinical studies most likely reflects its multiple sites of action. The profile of anticonvulsant activity may suggest among others the modulation of postsynaptic AMPA receptors’ function as well as the inhibition of glutamate release from the presynaptic neurons in the hippocampus (e.g., through the influence on SV2A protein of synaptic vesicles or inhibition of presynaptic voltage-gated Ca^2+^ channels) [73–78]. In the aim of determination of AS-1 interaction with other binding sites on sodium channels (beyond site 2, see Table 4), we studied its influence on sodium currents in rat prefrontal cortex pyramidal neurons using the patch clamp technique. The results obtained indicate that AS-1 does not interact with fast voltage-gated sodium channels at a concentration of 100 μM.

The ADME-Tox parameters evaluated for the compound AS-1 by *in vitro* methods included permeability, metabolic stability, DDIs, and hepatotoxicity. The PAMPA was used here to determine the passive diffusion of AS-1 through artificial membrane, which imitates the cellular one. The passive transport is a very important parameter to be determined at the early stage of drug discovery process, as it is the predominant mechanism for absorption of most commercial drugs [79]. As a result, AS-1 showed very good ability to passive diffusion in comparison to the highly permeable reference caffeine. The metabolic stability is another important parameter, since the structures that are highly active *in vitro* may be susceptible to metabolism in the body and not reach the molecular target [79]. In our study, no phase I metabolites were observed at the UPLC spectra after the 120-min incubation with HLMs, indicating an excellent metabolic stability of the compound AS-1. Many DDIs can occur when two drugs are co-administered and compete for the same enzyme. Most of the detected DDIs were mediated by the cytochrome P450 family of enzymes [79]. Thus, during this study, the effect of AS-1 on CYP3A4, 2D6, and 2C9 activity was investigated and compared to the respective inhibitors. Notably, the aforementioned CYP isoforms are responsible for the metabolism of approximately 70% of all marketed drugs [79]. No significant effect of AS-1 on CYP3A4 and CYP2D6 activity was observed, whereas moderate CYP2C9 inhibition was found at the highest used doses of 10 μM and 25 μM. The obtained results indicated a low risk of potential DDIs after potential drug co-administration with AS-1. In our previous study [12], AS-1 was incubated with HepG2 cells for 24 h and showed a weak, statistically significant toxic effect only at the highest used dose of 100 μM. To exclude the potential long-term hepatotoxic effect, AS-1 incubation time with cells was prolonged here up to 72 h. The slight but statistically significant effect on cells’ viability was observed only at the highest used dose of 100 μM and confirmed our previous data [12]. Thus, taking into account these both independent studies, the compound AS-1 showed a very low risk of potential hepatotoxicity.

## Conclusions

The results of the current studies proved that *N*-benzyl-(2,5-dioxopyrrolidin-1-yl)propanamide (AS-1) may be regarded as a novel wide-spectrum anticonvulsant agent. We showed that AS-1 produced antiseizure effect in the PTZ kindling model. Noteworthy, this molecule reveled also protection in the 6-Hz (44 mA) seizure test, which is recognized as an animal model of human drug-resistant seizures. The comprehensive pharmacological investigation described herein showed more potent anticonvulsant protection and clearly better safety profile for AS-1 *versus* VPA which belongs to the most frequently prescribed AEDs, as it is effective in case of different types of epilepsies. The isobolographic analysis indicated the supra-additive (synergistic) interaction of AS-1 and VPA in the PTZ-induced clonic seizures in mice, notably without increasing disturbances in the chimney, passive avoidance, and grip strength tests in mice. Thus, it is postulated that AS-1 + VPA in combination appeared to be beneficial and safe regarding the advanced clinical investigation. The high safety profile of AS-1 was also confirmed in the *in vitro* studies, as it showed no significant influence on the function of CYP3A4/CYP2D6 activity, only moderate inhibition of CYP2C9 at a concentration of 10 μM, and no hepatotoxic properties in HepG2 cells (concentration of 10 μM). Unfortunately, the *in vitro* binding and functional studies, as well as patch clamp technique, did not provide firm evidence on the plausible mechanism of action for AS-1.

## Electronic supplementary material

ESM 1 (DOCX 128 kb)

ESM 2 (PDF 193 kb)
