# Supplementary material for: N-Benzyl-(2,5-dioxopyrrolidin-1-yl)propanamide (AS-1) with Hybrid Structure as a Candidate for a Broad-Spectrum Antiepileptic Drug
Source: Neurotherapeutics. 2019 Sep 4;17(1):309–28. doi: 10.1007/s13311-019-00773-w (PMC7007424; doi:10.1007/s13311-019-00773-w)
Supplement: Supplementary file 1 — (DOCX 128 kb) [file 13311_2019_773_MOESM1_ESM.docx]

Supporting Information for

*N*-benzyl-(2,5-dioxopyrrolidin-1-yl)propanamide (AS-1) **with hybrid structure as a candidate for a broad spectrum antiepileptic drug**

**Table S1.** Physicochemical and spectral data for **AS-**1 S2

**Fig. S1** Effect of repeated treatment with **AS-1** on the percentage of the open arms entries and the percentage of the time spent in the open arms in the elevated plus maze test in PTZ-kindled mice S3

**Fig. S2** Effect of repeated treatment with **AS-1** on the immobility time in the forced swim test in PTZ-kindled mice S4

**Table S1.** Physicochemical and spectral data for **AS-1**

| Chemical name | ***N*-benzyl-(2,5-dioxopyrrolidin-1-yl)propanamide** (**AS-1**) |
| --- | --- |
| Physicochemical and spectral data | White solid, mp. 106.2**–**107.5ºC; UPLC (purity >99%): t_R_ = 3.88 min.; TLC: *R*_f_ **=** 0.52 (dichloromethane : methanol; 9 : 0.3; *v/v*);^1^H NMR (500 MHz, CDCl_3_) δ 1.41 (d, 3H, *J*=7.2 Hz), 2.61 (s, 4H), 4.26 (d, 2H, *J*=6.2 Hz), 4.57 (q, 1H, *J*=7.2 Hz), 7.13–7.36 (m, 5H), 8.32–8.36 (br. s, 1H); ^13^C NMR (126 MHz, CDCl_3_) δ 14.3, 28.1, 43.6, 49.5, 127.4, 127.5, 128.6, 137.8, 168.6, 176.8. ESI–MS: 261.3 (C_14_H_16_N_2_O_3_ [M+H]^+^). Anal. calcd for C_14_H_16_N_2_O_3_ (260.29): C: 64.60, H: 6.20, N:10.76; Found C: 64.72, H: 6.35, N: 10.69. |

**Fig. S1** Effect of repeated treatment with **AS-1** on the percentage of the open arms entries (panel A) and the percentage of the time spent in the open arms (panel B) in the elevated plus maze test in PTZ-kindled mice. **AS-1** and VPA were injected i.p. once daily for 33 days. Experimental groups consisted of 12–15 animals. Data are expressed as means + SEM. Statistical analysis: one-way ANOVA test.

**Fig. S2** Effect of repeated treatment with **AS-1** on the immobility time in the forced swim test in PTZ-kindled mice **AS-1** and VPA were injected i.p. once daily for 33 days. Experimental groups consisted of 12–15 animals. Data are expressed as means + SEM. Statistical analysis. one-way ANOVA followed by Bonferroni’s post hoc test: ** p < 0.01, *** p < 0.001 as compared to the control group; ^#^ p < 0.05 as compared to the to the kindled control group.
